# Lack of association of matrix metalloproteinase-3 gene polymorphism with susceptibility to rheumatoid arthritis: a meta-analysis

**DOI:** 10.1186/1471-2474-15-376

**Published:** 2014-11-18

**Authors:** Zhitao Feng, Guochao He, Zhuanghong Chen, Zhengzhi Wu, Juan Li

**Affiliations:** Department of Rheumatology, Nanfang Hospital, Southern Medical University, 1838 North of Guangzhou Road, Guangzhou, China; Department of Traditional Chinese Internal Medicine, School of Traditional Chinese Medicine, Southern Medical University, 1838 North of Guangzhou Road, Guangzhou, China; School of Medicine, Jinan University, 601 West of Huangpu Road, Guangzhou, China; Department of Orthopedic Surgery, Hunan Provincial Hospital of Traditional Chinese Medicine, 571 Renminzhong Road, Zhuzhou, China; Department of Orthopedic Surgery, Wuhan General Hospital of Guangzhou Military Command, 627 Wuluo Road, Wuhan, China; Shenzhen Institute of Geriatric Medicine, 1017 North of Dongmen Road, Shenzhen, China; The First Affiliated Hospital of Shenzhen University, 3002 West of Sungang Road, Shenzhen, China

**Keywords:** Rheumatoid arthritis, Matrix metalloproteinase, Polymorphism, Susceptibility, Meta-analysis

## Abstract

**Background:**

Epidemiological studies have investigated the association between matrix metalloproteinase-3(MMP-3) gene-1171 5A/6A polymorphism and rheumatoid arthritis (RA), but the results were inconsistent. To evaluate the specific relationship, we performed a meta-analysis to clarify the controversies.

**Methods:**

The relevant literatures dated to December 07th 2013 were retrieved from PubMed, EMBASE and the China National knowledge Infrastructure (CNKI) databases. The number of the alleles and genotypes for MMP-3 were obtained. Odds ratios (ORs) and 95% confidence intervals (CIs) were used to estimate the association between MMP-3 5A/6A promoter polymorphism and RA. All of the statistical analyses were conducted by STATA11.0 software.

**Results:**

A total of 6 case-control studies covering 1451 cases and 1239 controls were included in the final meta-analysis. There was no significant association between MMP-3 5A/6A promoter polymorphism and RA in all genetic models (for 6A versus 5A: OR = 1.19, 95% CI = 0.91-1.56, *P* = 0.203; 5A/6A versus 5A/5A: OR = 1.31, 95% CI = 0.89-1.92, *P* = 0.174; 6A/6A versus 5A/5A: OR = 1.78, 95% CI = 0.68-4.61, *P* = 0.238; the recessive model: OR = 1.48, 95% CI = 0.88-2.47, *P* = 0.141; and the dominant model: OR = 1.46, 95% CI = 0.71-3.00, *P* = 0.299). In the subgroup analysis by ethnicity, we obtained the similar results.

**Conclusions:**

We systematically investigate the association between MMP-3-1171 5A/6A polymorphism and RA susceptibility; however, the results show a lack of correlation. Considering the small sample size and the selection bias existed in some studies, further studies are needed to confirm the findings.

**Electronic supplementary material:**

The online version of this article (doi:10.1186/1471-2474-15-376) contains supplementary material, which is available to authorized users.

## Background

Rheumatoid arthritis (RA) is an autoimmune inflammatory disease and the etiology is still unknown. It is characterized by synovial inflammation and hyperplasia, autoantibody production, cartilage and bone destruction [1, 2]. The environment factor and genetic participate in mechanisms of RA [3, 4]. Recently, research has focused on the identification of genes that influence the susceptibility of this disorder. Therefore, analysis and identification of new genes associated with RA susceptibility is an important and meaningful challenge.

The clinical manifestations and outcomes of RA range from mild to severe polyarthritis with progressive destruction of cartilage and bone. Much of the destruction in RA is mediated by abnormal release of matrix metalloproteinase (MMPs) in synovium stimulated by persistent inflammation [5]. MMPs are a group of zinc-dependent endopeptidases, which can degrade every component of the extracellular matrix. In the synovial joint, MMPs are mainly secreted by fibroblasts, macrophages and chondrocytes. The expression of most MMPs is regulated at the transcription level by growth factors, hormones, and cytokines [6].

MMP-3(stromelysin 1) is considered to be the main MMP involved in cartilage degradation and the most widely studied member in RA. It has broader substrate specificity with activity against type II, III, IV, IX, X, XI collagens, proteoglycans, fibronectin and laminin. In addition, it can activate other MMPs such as MMP-1,-2, -9 and -13 [6, 7]. It has been reported that the serum and synovial fluid levels of MMP-3 are elevated in early and established RA patients, and are associated with diseased activity and /or joint destruction [8–11].

In recent years, a single nucleotide polymorphism (SNP) in the promoter sequence of the MMP-3 gene have been described [12–14], and this polymorphism may play an important role in regulating the MMP-3 gene expression [15]. In human, the MMP-3 gene is located at the long arm of chromosome 11 (11q22.3) [16], and the promoter region of MMP-3 is characterized by 5A/6A promoter polymorphism at position of -1171(rs3025082), in which one allele has six adenosines(6A) and the second has five adenosines(5A) [12], while the 6A allele has about half the promoter strength of the 5A allele [15].

The association between MMP-3 gene polymorphism and RA susceptibility has been investigated, but the results between studies are either inconsistent or lack strength owing to small sample sizes. Therefore, the purpose of this study was to ascertain whether polymorphism in the promoter region of MMP-3 gene was associated with RA susceptibility.

## Methods

### Publication search

This meta-analysis followed the preferred reporting items for Meta-analysis Of Observational Studies in Epidemiology (MOOSE) group [17]. The PubMed, Embase and Chinese National Knowledge Infrastructure (CNKI) databases were searched (updated to Dec 07th, 2013) with terms ‘Matrix Metalloproteinase 3’, ‘stromelysin 1’, ‘MMP-3’, ‘rs3025058’, ‘polymorphism’, ‘Genome-Wide Association Study’, ‘arthritis, rheumatoid’ and ‘RA’, as both medical subject heading (MeSH) terms and text words to find all papers that had studied the association of MMP-3 5A/6A SNP with RA. Manual search of references from original research or review articles was performed to identify additional studies. No language and time restrictions were applied.

### Inclusion criteria

Studies were included if they complied with all the following criteria: (a) case-control study on the association of MMP-3 5A/6A promoter polymorphism with RA; (b) sufficient published data for estimating the odds ratio (OR) with 95% confidence interval (CI); (c) For multiple publications reporting on the same data or overlapping data, the largest or most recent publication was selected [18].

### Exclusion criteria

Studies were excluded if: (a) containing overlapping data; (b) genotype distribution of the control population is not in Hardy–Weinberg equilibrium (HWE); and (c) studies in which family members had been studied because their analysis is based on linkage considerations.

### Data extraction

Study selection was carried out independently by two investigators (Feng and He) according to the inclusion and exclusion criteria listed above. The following data were extracted from eligible studies: the first author's name, year of publication, country of origin, ethnicity of the studied population, total numbers of cases and controls, numbers of cases and controls with different genotypes respectively. Furthermore, evidence of Hardy-Weinberg equilibrium (HWE) was collected. Data were extracted independently by two investigators (Feng and He), and consensuses were reached on all items. A third investigator (Juan Li) was to adjudicate any disagreement if consensus could not be reached.

### Quality score assessment

The quality of included studies was evaluated independently by two authors (Feng and He) of this article according to the Newcastle–Ottawa Scale (NOS) for case-control studies [19]. The NOS ranges between zero (worst) and nine stars (best). Disagreement was resolved by discussion. A third investigator (Juan Li) was to adjudicate any disagreement if it was necessary.

### Statistical methods

Fisher's exact test was used to assess deviation from HWE in the control group according to genotypes. Crude OR with their 95% CI was estimated and used to assess the strength of association between MMP-3 5A/6A promoter polymorphism and RA. The pooled OR was calculated respectively for allelic effect of 6A versus 5A, homozygote comparison of 6A/6A versus 5A/5A, recessive model (6A/6A versus 5A/5A +5A/6A) and dominant model (6A/6A +5A/6A versus 5A/5A). The significance of the pooled OR was determined by the Z-test. (*P* ≤0.05 was considered representative of statistical significance).

Heterogeneity among studies was examined using Cochran's Q statistic and the *I*^2^ statistic (*P* <0.10 and *I*^2^ > 50% indicated the evidence of heterogeneity) [20]. If there was no statistical heterogeneity among studies, the fixed-effect model was used; otherwise the random-effect model was used [21]. Subgroup analysis was performed by ethnicity. Sensitivity analysis was determined by deleting each single study in each meta-analysis to reflect the influence of the individual data-set to the pooled OR. An estimate of potential publication bias was carried out by Begg's funnel plot and Egger's regression test [22]. All of the statistical analyses were conducted by STATA11.0 software (Stata Corporation, College Station, TX, USA). (*P* ≤0.05 was considered significant). Our study followed the PRISMA guidelines (Additional file 1).

## Results

### Characteristics of included studies

**Figure 1:**
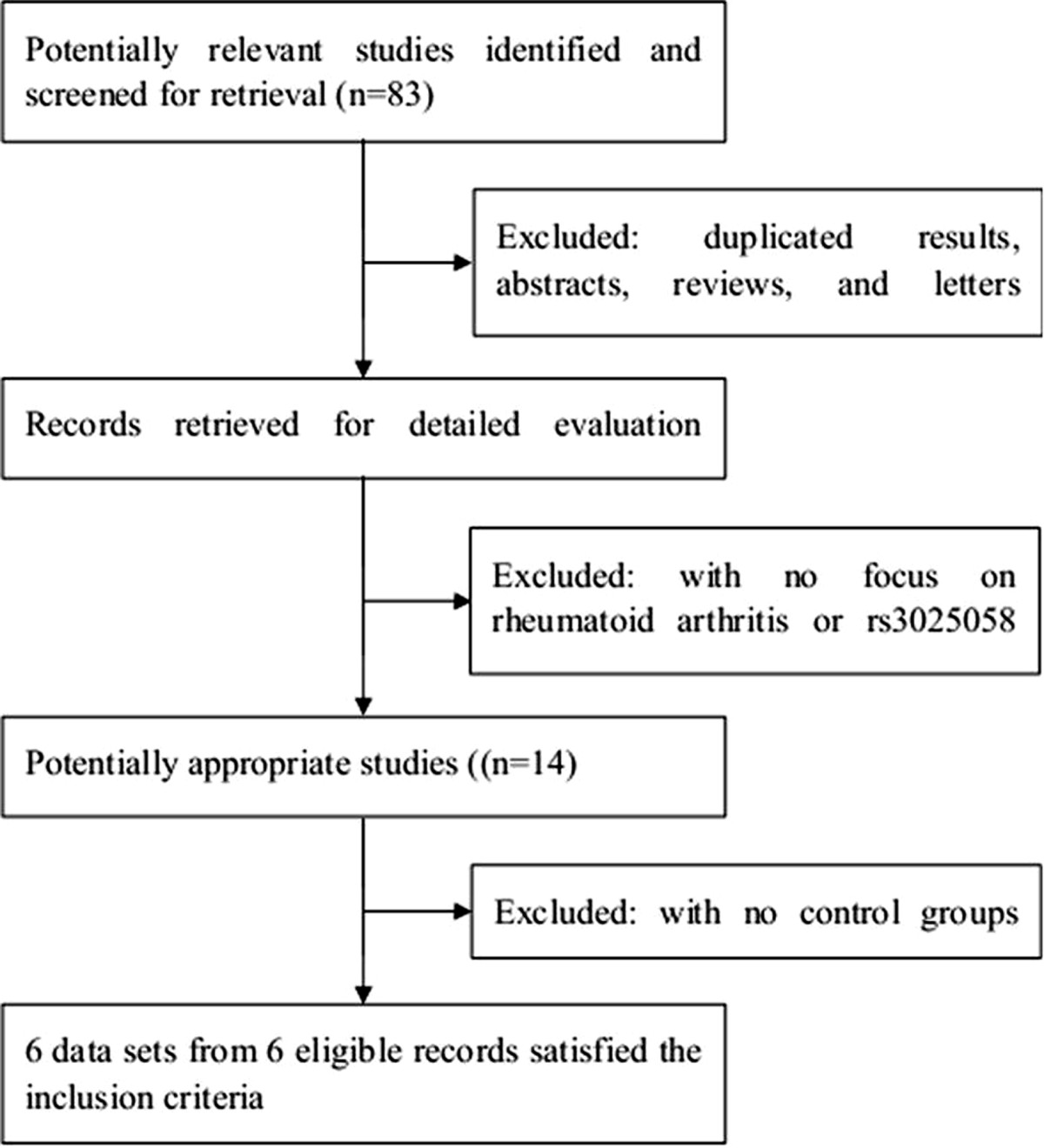
**Flow diagram of selected of studies and specific reasons for exclusion from the meta-analysis.**

The study selection process is detailed in Figure 1. Based on our search strategy, 83 relevant studies were achieved by identification through systematic searches and a review of references. 28 eligible studies were retrieved for detailed evaluation. During the procedure of data extraction, 14 articles were excluded because they were not focused on RA or rs3025058. In addition, 8 records were because they did not have control groups. Finally, a total of 6 case-control studies including 1451 cases and 1239 controls in 6 articles met our inclusion criteria reported the association between MMP-3 5A/6A promoter polymorphism and RA [23–28]. The characteristics of selected studies were summarized in Table 1. Of the ethnicity among all studies, there were five in Caucasian [23–25, 27, 28] and one in Asian [26]. The distribution of genotypes did not deviate from HWE in the controls group of all included studies. The NOS results showed that the average score was 7.67, which indicated that the methodological quality was generally good.

**Table 1:** **Characteristics of studies included in this meta-analysis**

Author year	Ethnicity	HWE ( *P*)	Score	Sample size (case/control)	Case (%)					Control (%)				
					5A	6A	6A/6A	5A/6A	5A/5A	5A	6A	6A/6A	5A/6A	5A/5A
Abd-Allah 2012	Caucasian (Egypt)	Y (0.986)	8	100/100	88 (44.0)	112 (56.0)	33 (33.0)	46 (46.0)	21 (21.0)	128 (64.0)	72 (36.0)	13 (13.0)	46 (46.0)	41 (41.0)
Constantin 2002	Caucasian (France)	Y (0.213)	8	102/126	98 (48.0)	106 (52.0)	27 (26.5)	52 (51.0)	23 (22.5)	111 (44.0)	141 (56.0)	36 (28.5)	69 (54.8)	21 (16.7)
Dörr 2004	Caucasian (Germany)	Y*	8	308/110	306 (49.6)	310 (50.4)	NM	NM	NM	103 (46.8)	117 (53.2)	NM	NM	NM
Rodrigue 2006	Caucasian (Spain)	Y*	7	550/652	556 (50.5)	544 (49.5)	NM	NM	NM	674 (51.7)	630 (48.3)	NM	NM	NM
Scherer 2010	Caucasian (Brazil)	Y (0.130)	8	110/100	71 (32.3)	149 (67.7)	52 (47.3)	45 (40.9)	13 (11.8)	90 (45.0)	110 (55.0)	34 (34.0)	42 (42.0)	24 (24.0)
Zhou 2007	Asian (China)	Y (0.150)	7	281/151	65 (11.6)	497 (88.4)	224 (79.7)	49 (17.4)	8 (2.8)	36 (11.9)	266 (88.1)	119 (78.8)	28 (18.5)	4 (2.7)

HWE: Hardy–Weinberg equilibrium, Y: yes, N: no. *confirmed in original study, Score: The Newcastle-Ottawa Scale for Case-control Study, NM: none mention.

### Quantitative synthesis

The association between MMP-3 5A/6A promoter polymorphism and RA was investigated in six studies with a total of 1451 RA cases and 1239 controls. Given that significance between-study heterogeneity existed in overall comparisons except 5A/6A versus 5A/5A (for 6A versus 5A: *I*^2^ = 76.4%, *P*_h_ = 0.001; 5A/6A versus 5A/5A: *I*^2^ = 50.9%, *P*_h_ = 0.106; 6A/6A versus 5A/5A: *I*^2^ = 78.6%, *P*_h_ = 0.003; the recessive model: *I*^2^ = 68.6%, *P*_h_ = 0.023; and the dominant model: *I*^2^ = 71.3%, *P*_h_ = 0.015), we used fixed-effect model for comparison 5A/6A versus 5A/5A and used the random-effect model for others. Overall, there was no significant association between MMP-3 5A/6A promoter polymorphism and RA (for 6A versus 5A: OR = 1.19, 95% CI = 0.91-1.56, *P* = 0.203; 5A/6A versus 5A/5A: OR = 1.31, 95% CI = 0.89-1.92, *P* = 0.174; 6A/6A versus 5A/5A: OR = 1.78, 95% CI = 0.68-4.61, *P* = 0.238; the recessive model: OR = 1.48, 95% CI = 0.88-2.47, *P* = 0.141; and the dominant model: OR = 1.46, 95% CI = 0.71-3.00, *P* = 0.299). The main results of meta-analysis were shown in Table 2.

**Table 2:** **Results of quantitative synthesis involved the association between MMP-3 5A/6A polymorphisms and RA**

Comparison model	Overall					Caucasians			
	Sample size (case/control)	OR (95% CI)	*P*_*h*_	*P*	*P*_*r*_	Sample size (case/control)	OR (95% CI)	*P*_*h*_	*P*
6A vs 5A	2902/2478	1.19(0.91-1.56)*	0.001	0.203	0.461	2340/2176	1.23(0.89-1.68)*	0.000	0.207
5A/6A vs 5A/5A	257/275	1.31(0.89-1.92)	0.106	0.174	0.824	200/243	1.37(0.68-2.76)*	0.058	0.373
6A/6A vs 5A/5A	401/292	1.78(0.68-4.61)*	0.003	0.238	0.835	169/169	2.11(0.66-6.67)*	0.002	0.209
Dominant model	593/477	1.46(0.71-3.00)*	0.015	0.299	0.766	312/326	1.61(0.68-3.80)*	0.008	0.274
Recessive model	593/477	1.48(0.88-2.47)*	0.023	0.141	0.257	312/326	1.69(0.84-3.40)*	0.021	0.142

Dominant model: 6A/6A +5A/6A versus 5A/5A, Recessive model: 6A/6A versus 5A/5A +5A/6A, OR: odds ratio, CI: confidence interval, Ph: P -value of Q-test for heterogeneity test, P: P -value of Z-test for significance of the pooled OR, Pr: P -value of Egger's regression test for publication bias, *Significant heterogeneity: the random-effect model was chosen to summarize the result.

In the subgroup analysis by ethnicity, we only analyzed the Caucasians as just one study involved in Asians populations. We obtained similar results that no significant association was found in all genetic models. The results of subgroup analysis quantitatively illustrated that MMP-3 5A/6A promoter polymorphism did not associate with RA. The main results of subgroup analysis were shown in Table 2.

### Sensitivity analysis and Publication bias

**Figure 2:**
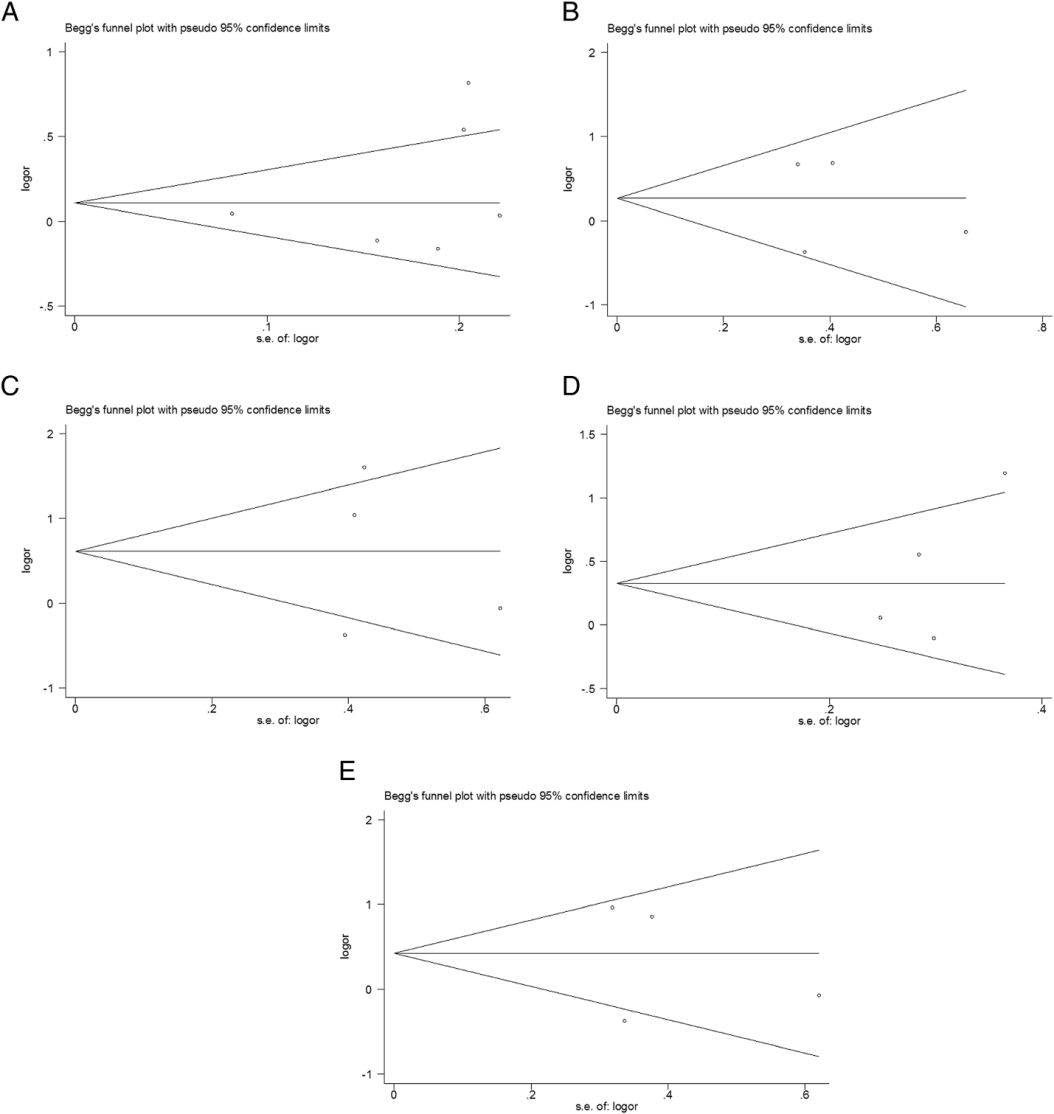
**Funnel plots of the association between MMP-3-1171 5A/6A polymorphism and RA. A** (alleles model: 6A vs 5A), **B** (alleles model:5A/6A vs 5A/5A), **C** (alleles model: 6A/6A vs 5A/5A), **D** (Recessive model: 6A/6A versus 5A/5A +5A/6A) and **E** (Dominant model: 6A/6A +5A/6A vs 5A/5A).

Sensitivity analyses were preformed to assess the stability of the results. The result did not change when a single study involved in the meta-analysis was deleted each time. The shapes of the Begg's funnel plots did not reveal any evidence of obvious asymmetry in all genetic models (Figure 2). Meanwhile, the results of Egger's regression test still did not provide any evidence of publication bias (*P* = 0.461 for 6A versus 5A, *P* = 0.824 for 5A/6A versus 5A/5A, *P* = 0.835 for 6A/6A versus 5A/5A, *P* = 0.257 for recessive model and *P* = 0.766 for dominant model, respectively) (Table 2).

## Discussion

RA is characterized by chronic inflammation of synovial tissue and progressive destruction of cartilage and bone. Progressive joint destruction is one of the strongest predictors of long-term outcome and disability in RA [29]. Synoviocytes produce a wide range of proinflammatory cytokines such as interleukin 1(IL-1), IL-6 and tumour necrosis factor (TNF) which stimulate osteoclast like cells to secrete proteolytic enzymes such as MMPs. IL-1 stimulates synoviocytes and chondrocytes to release MMPs such as MMP-3 that degrade collagen, resulting in extracellular matrix degradation ultimately leading to cartilage and bone loss [30, 31].

MMP-3 is believed to play a pivotal role involving in joint destruction in RA, and there is a common polymorphism in the promoter sequence of the MMP-3 gene [12, 13], which may be correlated with RA susceptibility. Although large-scale genome wide association studies (GWAS) reveal a number of SNP markers that reproducibly associate with RA susceptibility [3], substantially improving our understanding of the genetic component of disease susceptibility, however, hundreds of common risk alleles are likely to exist but remain undiscovered to date owing to the limited power of current GWAS [4]. And the association between MMP-3 gene polymorphism and RA was poorly understood.

Currently, more and more evidences showed that 5A/6A polymorphisms in the MMP-3 gene promoter were presumably associated with susceptibility and severity of RA. Constantin et al. found that the MMP-3 6A/6A genotype was associated with the highest total radiographic damage score (TDS) both at baseline and after a 4-year follow-up and with the highest progression of the TDS over the 4 years of follow-up in patients with early RA, but not RA susceptibility. And they also showed the serum concentration of MMP-3 did not differ between the three MMP-3 genotypes [23]. Likewise, Mattey et al. showed established RA patients homozygous for the MMP-3 6A allele had more radiographic damage than those with other genotypes, but that patients with the 6A/6A genotype also had more functional impairment and higher serum proMMP-3 levels [32]. In Japanese patients Tsukahara et al. found the effect of the 6A allele on increasing level of serum MMP-3, no significant effect of the polymorphism was found on the disease activity or severity of RA, though a trend of an effect of 5A allele on the Sharp/van der Heijde score was observed [33]. Interestingly, Scherer et al. observed that the 6A allele was associated with higher RA susceptibility, but RA patients homozygous for 6A allele have significant lower frequency of extra-articular manifestation and of rheumatoid nodules than patients carrying 5A allele [27]. In the study of Abd-Allah et al. also found that there were significant associations between MMP-3 (-1171 5A/6A) polymorphism and susceptibility to RA. The 6A/6A genotype and 6A allele were significantly more frequent in the patients with RA and than in the control group. There was also an association between MMP-3 5A/6A polymorphism and the severity in RA patients. But, they found that there were no significant association between the MMP-3 levels and the allelic variants of MMP-3 polymorphism [28]. Meanwhile, Nemec et al. revealed RA patients with 5A allele presented more progressive radiographic joints damage, but with the 6A/6A genotype had lower risk to develop erosive RA [34]. Ye et al. reported that RA patients with the MMP-3 5A/5A genotype were also associated with higher Steinbrocker index and health assessment questionnaire [35]. However, the results from Rodriguez-Lopez et al. showed that 5A allele frequency did not disclose significant differences between RA patients and controls [25]. Even more peculiar, Dörr et al. found no association between MMP-3 polymorphism and the susceptibility or radiographic damage, and the plasma concentrations of MMP-3 were not significantly different between patients groups with defined MMP alleles [24].

From the above it can be seen that the conflict results and small sample size are too underpowered to detect a possible effect of the MMP-3 gene polymorphism on RA. Thus, we conducted this meta-analysis to better understand the association between MMP-3 gene polymorphism and susceptibility of RA.

To the best of our knowledge, this is the first meta-analysis to investigate the association of MMP-3 gene polymorphism with RA, and the influence of this gene polymorphism on RA susceptibility in different ethnic populations. In this meta-analysis, a total 1451 RA patients and 1239 controls were analyzed to provide overall assessment of the association between MMP-3 5A/6A polymorphism and RA susceptibility. The results manifested that there was no association between MMP-3 5A/6A polymorphism and RA susceptibility. Neither allele frequency nor genotype distribution was significantly associated with susceptibility to RA. Considering the ethnic may influence the consequences, subgroup analyses were performed to further investigate the potential association. However, the similar results were observed in Caucasians.

It is not surprising that our study failed to detect any connection of the MMP-3 polymorphism with the susceptibility to RA. There are several reasons for this phenomenon, first of all, RA is a complex disease and multiple genes, different genetic backgrounds, and different environmental factors lead to the development of RA. Secondly, the MMP-3 5A/6A polymorphism is in linkage disequilibrium with MMP-1 1G/2G which is linked to RA [24]. Thirdly, some other as-yet unidentified genes might conceal the influence of the alleles. Therefore, our findings suggest that further investigations are required before we are able to determine the association between the MMP-3 5A/6A polymorphism and RA.

Moreover, the present study has some limitation should be discussed. First, significance between-study heterogeneity was observed in most comparisons. It may affect the precision of results although we use random-effect model to pool ORs. The heterogeneity may attribute to the confounding factors due to case definition, sample size and methods of genotyping. Although we conducted sensitivity analysis, the heterogeneity was still observed. Second, our meta-analysis included data from Caucasian and Asian, thus our study should be optimized by larger scale of populations. Third, lack of the original data of available studies limited our further evaluation of potential interactions, such as age, gender, environmental factors. Forth, due to different assessment methods of joints destruction, we did not investigate the association between MMP-3 gene polymorphism and RA severity. Finally, some inevitable publication bias may exist in the results, although neither the Begg's funnel plots nor Egger's regression test indicated obvious publication bias in our meta-analysis.

## Conclusions

Our meta-analysis demonstrate that a lack of MMP-3 gene polymorphism with susceptibility to RA. Nevertheless, increasing evidences manifest that this polymorphism may correlate with severity of RA. Thus, further studies in large cohorts of RA and in different populations are necessary to elucidate the association between MMP-3 -1171 5A/6A polymorphism and RA.

## Electronic supplementary material

Additional file 1:PRISMA checklist.(DOC 70 KB)

Below are the links to the authors’ original submitted files for images.

Authors’ original file for figure 1

Authors’ original file for figure 2

Authors’ original file for figure 3

Authors’ original file for figure 4

## Abbreviations

RA: Rheumatoid arthritis
MMP: Matrix metalloproteinase
HWE: Hardy-Weinberg equilibrium
SNP: Single nucleotide polymorphism
GWAS: Genome Wide Association Studies.
