# Supplementary figures and images for: Lack of association of matrix metalloproteinase-3 gene polymorphism with susceptibility to rheumatoid arthritis: a meta-analysis
Source: BMC Musculoskelet Disord. 2014 Nov 18;15:376. doi: 10.1186/1471-2474-15-376 (PMC4247749; doi:10.1186/1471-2474-15-376)

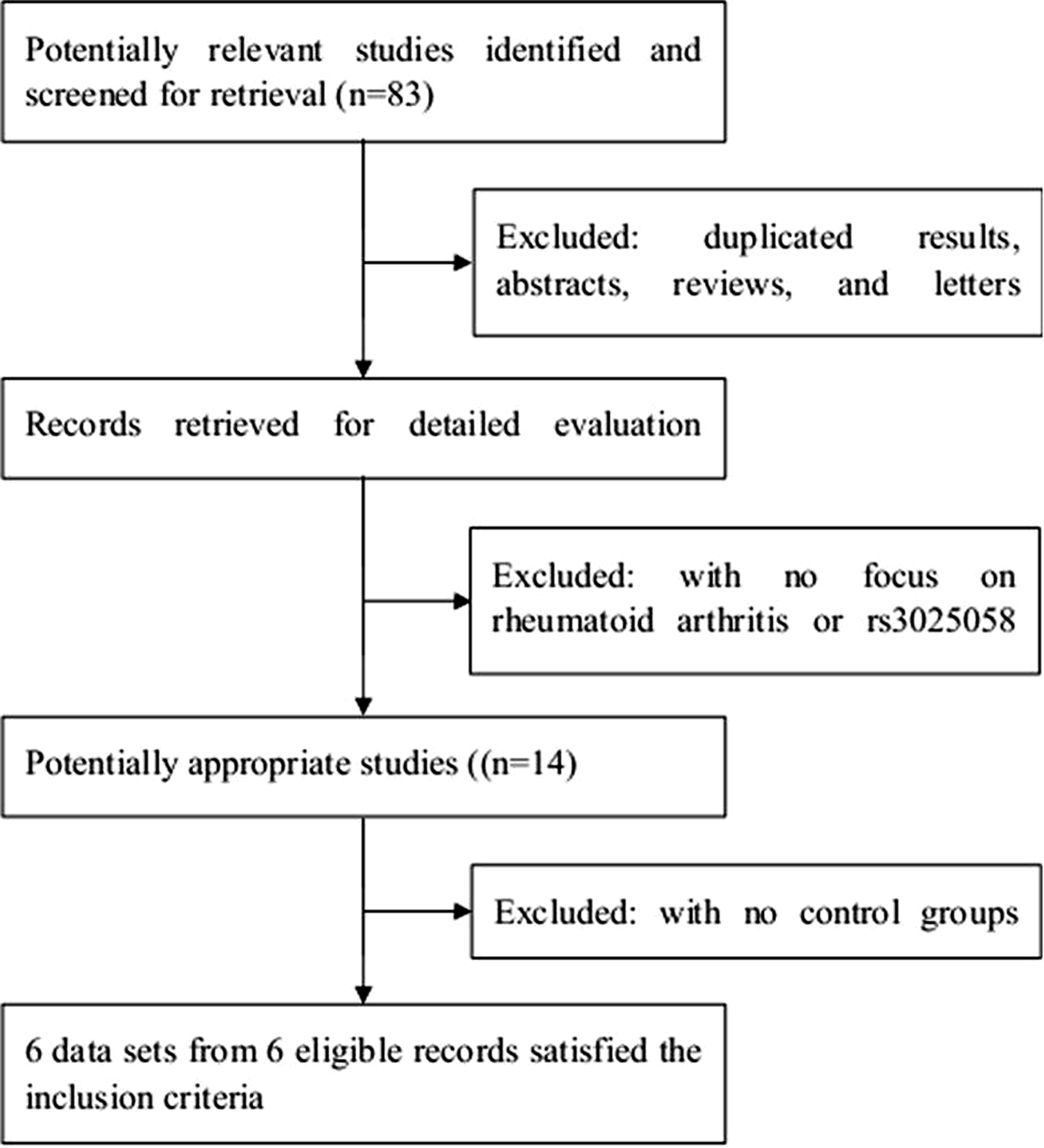

Supplement: Supplementary file 2 — Authors’ original file for figure 1 [file 12891_2014_2322_MOESM2_ESM.tif]

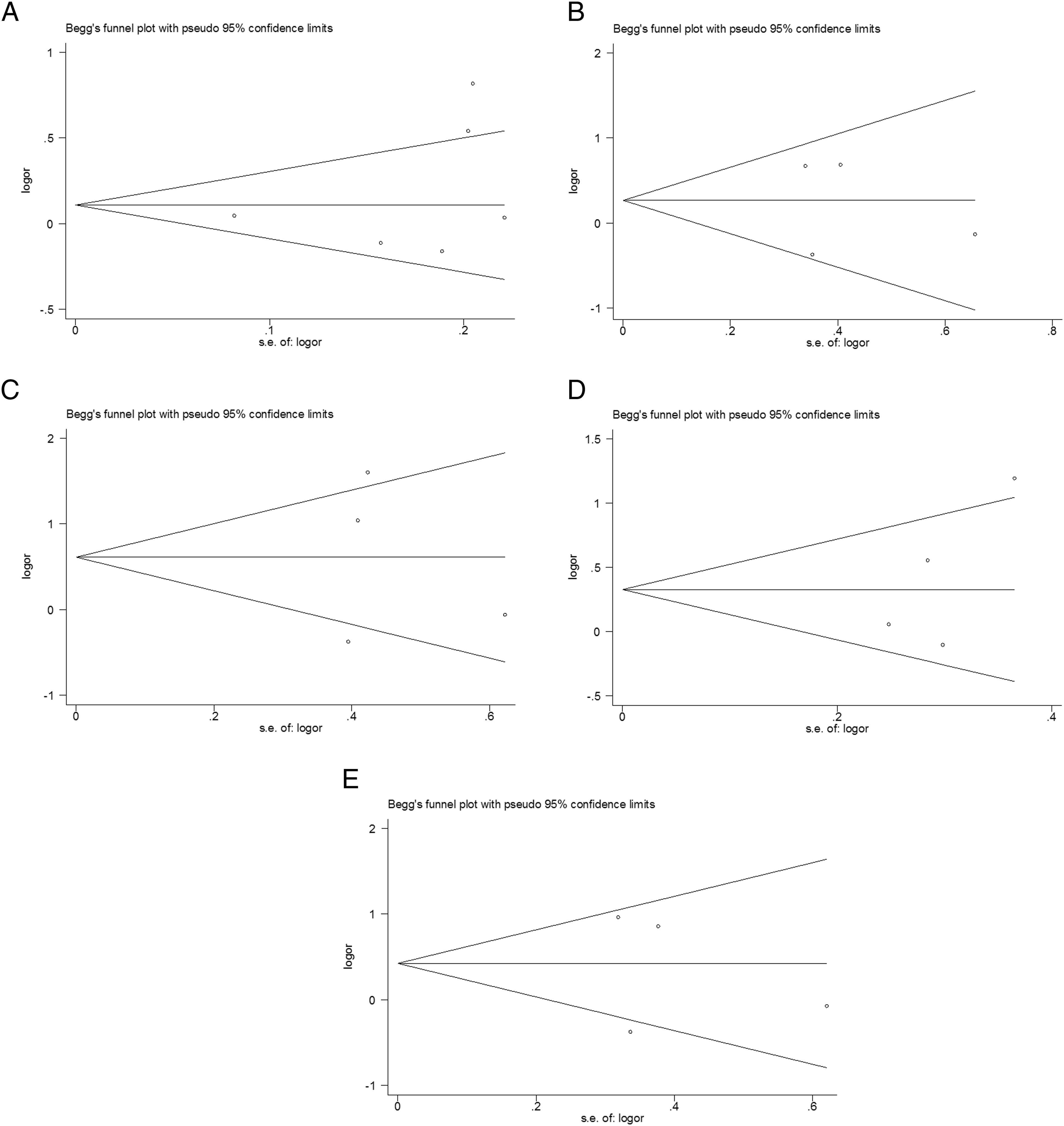

Supplement: Supplementary file 3 — Authors’ original file for figure 2 [file 12891_2014_2322_MOESM3_ESM.tiff]

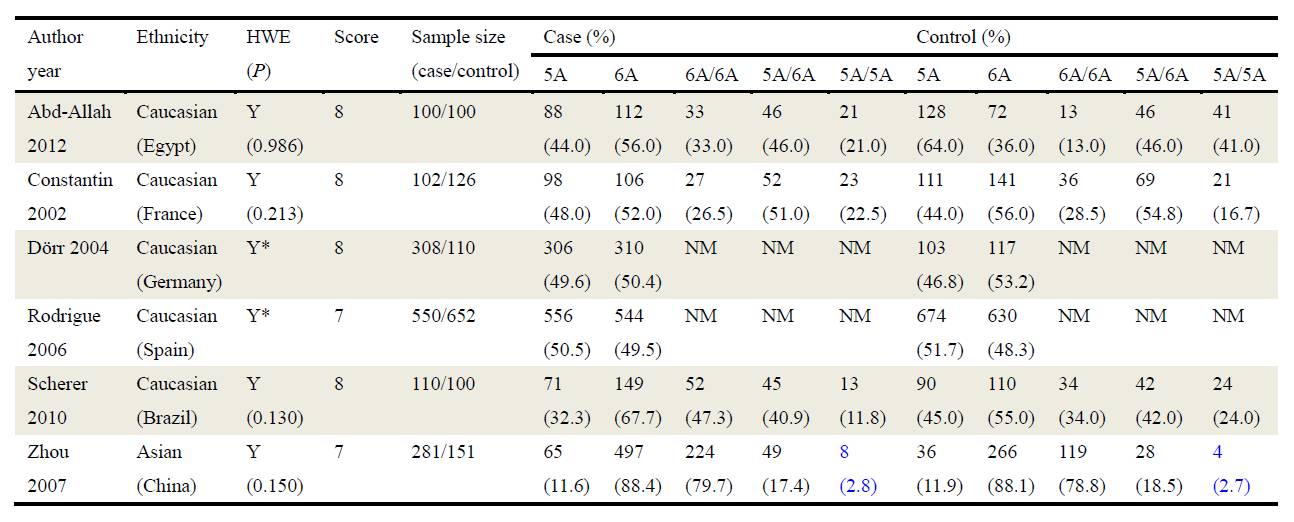

Supplement: Supplementary file 4 — Authors’ original file for figure 3 [file 12891_2014_2322_MOESM4_ESM.jpeg]

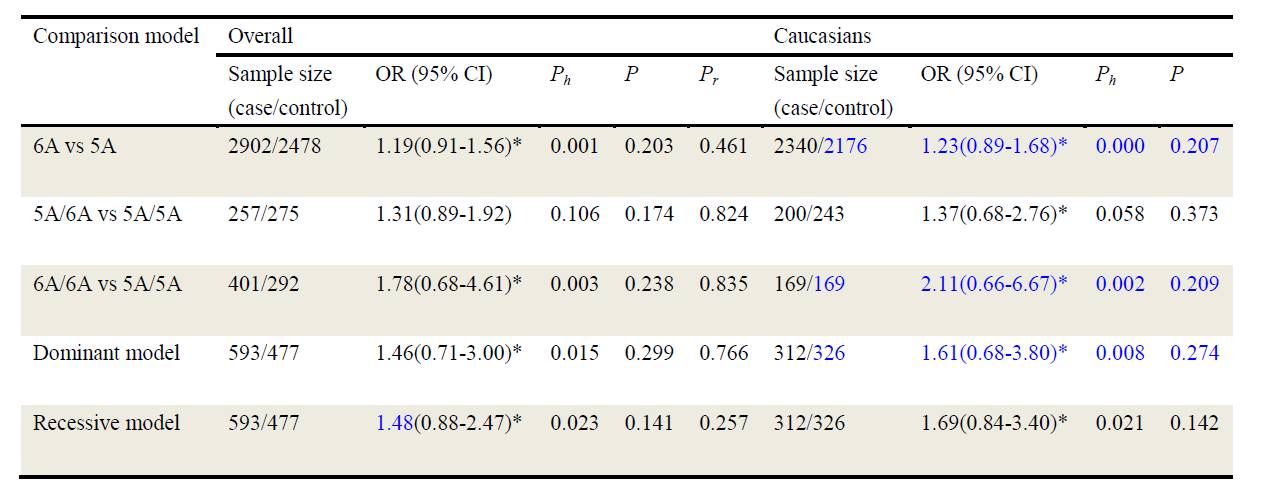

Supplement: Supplementary file 5 — Authors’ original file for figure 4 [file 12891_2014_2322_MOESM5_ESM.jpeg]
